# Non-hospital occupational blood exposure accidents: A nine-year retrospective analysis of post-exposure HIV, hepatitis B and C risk management in the Netherlands

**DOI:** 10.1017/S0950268826101319

**Published:** 2026-03-27

**Authors:** Elfi E.H.G. Brouwers, Henriëtte L.G. ter Waarbeek, Carlijn Somers, Nicole H.T.M. Dukers-Muijrers, Casper D.J. den Heijer, Christian J.P.A. Hoebe

**Affiliations:** 1 Department of Health Policy and Development, Living Lab Public Health Mosa (AWPG Mosa), South Limburg Public Health Service, 6400 AA Heerlen, The Netherlands; 2 Department of Social Medicine, Care and Public Health Research Institute (CAPHRI), Faculty of Health, Medicine and Life Sciences, Maastricht University, 6200 MD Maastricht, The Netherlands; 3 National Institute for Public Health and the Environment, RIVM, 3720 BA Bilthoven, The Netherlands; 4 Department of Health Promotion, Care and Public Health Research Institute (CAPHRI), Faculty of Health, Medicine and Life Sciences, Maastricht University, 6200 MD Maastricht, The Netherlands; 5 Department of Medical Microbiology, Infection Prevention and Infectious Diseases, Care and Public Health Research Institute (CAPHRI), Faculty of Health, Medicine and Life Sciences, Maastricht University Medical Centre (MUMC+), 6202 AZ Maastricht, The Netherlands

**Keywords:** blood-borne virus (BBV), blood exposure accidents, hepatitis B, hepatitis C, HIV, infectious diseases, needle stick injuries (NSI), occupational health, post-exposure risk assessment, public health, vaccination

## Abstract

Occupational blood exposure accidents (OBEAs) pose significant risks to healthcare workers, potentially exposing them to hepatitis B (HBV), hepatitis C (HCV), and HIV. While most research focuses on hospital settings, this study assessed OBEA management in non-hospital contexts. Although our data predate the COVID pandemic, findings remain highly relevant, especially for healthcare professionals working outside hospital settings. A retrospective analysis of OBEA registry data (2006–2014) was conducted in a southern Dutch region. Data included demographics, profession, workplace, injury type, source status (HBV, HCV, HIV), risk assessment, post-exposure measures, and lab results. Chi-square and t-tests were applied. In total, 975 OBEA were reported. Among nurses, medical assistants, students, and housekeeping staff, subcutaneous needles (51–67%) and lancets (25%) were common exposure sources. Police officers mainly reported biting (26%), scratching, or spitting (70%). HBV vaccination coverage ranged from 18% (housekeeping) to over 90% (nurses, police). Post-exposure measures were taken in 52% of cases. High-risk exposures (43%) mainly affected ambulance staff, sterilization workers, police, and dentists. Sources were tested in 85% of high-risk cases: 1.4% were HBV positive, 2% HCV positive, and 1.1% HIV positive. No seroconversions occurred. Results stress the need for better HBV vaccination coverage, targeted prevention, and prompt OBEA reporting outside hospital settings.

## Introduction

Both within and outside hospital settings, workers may acquire occupational injuries which bring a potential risk of occupational blood exposure accidents (OBEAs) [1–3]. The most common blood-borne viruses are hepatitis B virus (HBV), hepatitis C virus (HCV), and human immunodeficiency virus (HIV). According to literature, most research is done in the hospital setting, while outside the hospital scarce data are available.

The importance of non-hospital studies is increasingly evident in the context of the World Health Organization’s (WHO) 2030 goal to eliminate hepatitis and HIV. In this perspective, it is worrying that only little reduction in needle stick injuries (NSI) has been observed [4, 5], despite the implementation of EU regulations, including legislative safety needles. Hence, OBEAs remain a significant concern, including those occurring outside hospital settings [1].

In healthcare settings, the rate of HBV transmission relating to a percutaneous injury is estimated to be at an average of up to 30%: for HCV this is of 1–3% and for HIV 0.3% [6]. Biting injuries seem to have only a low risk of HBV and HCV infection, whereas for a splash accident, this is thought to be lower than that of other injuries [7]. For HIV, the transmission risk after a single mucocutaneous exposure is probably less than one in thousand (0.1%) [6]. There is no evidence that spitting incidents where no blood is present leads to acquisition of HIV [8, 9].

Prevention of OBEA and appropriate immediate management following such accidents remain crucial to prevent acquired infections. Timely intervention is essential to reduce the impact of these viruses, as infections can lead to long-term health consequences. Vaccination against HBV is highly effective in preventing transmission [10]. Moreover, highly effective treatments for HBV, HCV, and HIV are now available, capable of managing or even curing these infections.

WHO estimated that sharp injuries cause about 66,000 HBV, 16,000 HCV, and 2000–5,000 HIV infections among health care workers (HCW) per year worldwide. Furthermore, NSI contribute to 39%, 37%, and 4.4% of HCV, HBV, and HIV occupational infections for HCW worldwide respectively [11, 12].

In 2009, a European framework for workplace safety was established [13]. This agreement would contribute in a major way towards the improvement of working conditions of millions of HCW in the EU and beyond. A 2021 review later found a high prevalence of occupational exposure to NSI among HCW and suggests the need to improve occupational health and safety services [14].

Especially in lower-middle socio-demographic index (SDI) countries, pooled prevalence of OBEA for all HCW was higher (61.0%), but still substantial in high SDI (37.6%) and low SDI countries (41.6%), according to a recent systematic analysis of 83 studies. When assessing regions, the highest prevalence occurred in Southeast Asia at 58.2% [15].

In the Netherlands, healthcare professionals are not universally screened for HBV, HCV, or HIV, and HBV vaccination is not legally mandatory, although employers are required under the Working Conditions Act (Arbowet) [16] to offer vaccination; since 2011, HBV vaccination has also been offered to all newborns.

The Netherlands reported annually an estimated 13,000–15,000 blood-borne accidents, of which 95% originated from the healthcare sector [17]. The majority of data and publications on OBEAs pertain to hospital staff, even though OBEA occur frequently outside the hospital [18]. HCW in long-term care facilities and home care are at significant risk of these accidents [19], but detailed information about its circumstances is lacking.

However, given the aging population, it is likely that more people will be working in long-term care facilities and home care. A comprehensive understanding of OBEA is essential for providing sound recommendations regarding prevention and management as well as the necessary education for employees. Moreover, the international migration of healthcare workers, coming from non-western countries with sometimes high prevalence of blood-borne viruses, is becoming a means of maintaining service quality in long-term care facilities and home care in many high-income countries [20, 21]. In the Netherlands, 19% of the HCW in home care, 17% in care and nursing, 16% in mental healthcare, and 12% in care for the disabled have a migrant background already [22].

To our knowledge, only a few studies included OBEA data on non-hospital settings. One study, conducted in Belgium, provided detailed data on the causes and management of OBEA specific in long-term care facilities, concluding, among other findings, that insulin pens play a prominent role in NSI among nurses and nurse assistants [23]. However, this study did not focus on all non-hospital settings. A Dutch study focused on the practical management of OBEAs demonstrated improvement in the management and care of workers experiencing OBEA in both the hospital and non-hospital setting when incidents were reported to an expert 24 h-counselling centre. While many different workplaces and professions were included, there was no detail about the types of injuries that occurred in different settings. Schneeberger et al. concluded that more research is needed to explore OBEA risks and to design tailored preventive measures [24, 25]. To address this knowledge gap, we conducted a 9-year retrospective descriptive analysis of nearly 1,000 registered OBEA cases occurring outside hospital settings across various professional groups.

The aim of our study was to gain a deeper understanding of the occurrence, circumstances, risks, risk groups, and post-exposure measures of work-related blood exposure accidents outside the hospital to inform recommendations on the management of OBEA for practice and policy.

## Method

Under occupational health and safety legislation, employers in the Netherlands are obligated to protect the health of their employees. This includes a policy for OBEA. The management of an OBEA can be done by the care institution itself, the local PHS, or by another (national) organization that handles these accidents. Employees should report an OBEA as soon as possible to their employer. Guidelines on reporting and management are part of the employer’s OBEA policy and are based on the Dutch national guideline on needle stick accidents [26].

### Data collection

All non-hospital employees who reported an OBEA to Public Health Service (PHS) South-Limburg (serving a population of about 610.000 inhabitants) during the time period January 2006 to December 2014 were included in this study. Researchers only had access to anonymized data; they could not use any personal data, as these had been removed.

For each OBEA notification, a standardized accident registration form was used to collect systematic data on sex, age and profession of the employee, hepatitis B vaccination status, workplace, times of accident and reporting, type of injury, and causal device. Risk assessment and post-exposure measures taken were also registered. Employee’s serum test results (reference serum and follow-up serum) and source’s personal data and serostatus on HBV, HCV, and HIV were collected if deemed necessary (and possible) according to the Dutch national guideline [26]. See Supplementary Appendix 1.

### Interventions: Guideline on high-risk and low-risk blood exposure accidents

Each OBEA was handled by a PHS public health nurse or communicable disease consultant, following the Dutch national guideline. This guideline includes a risk assessment methodology based on the nature of the injury and its post-exposure management in practice to protect the exposed individual. OBEAs are categorized as either high risk or low risk based on the volume of blood transmitted and type of OBEA. Exposures with no risk of transmission are not part of the categorization and will not be notified to PHS. For low-risk accidents with no visible blood or blood on superficially damaged skin, measures to prevent HBV must be looked at: immunization of non-immune employees, either not (fully) vaccinated or non-responder (HBV anti-bodies <10 IU/L). For high-risk injuries involving a significant amount of blood exposure and a corresponding risk of transmission (e.g. percutaneous injuries or blood on mucous membrane), not only measures to prevent HBV but also to prevent/monitor HCV and HIV infection need to be considered. These consist of reference and follow-up serum testing on both HCV and HIV antibodies right after the accident and after 3 months and on HCV antibodies after 6 months, as well as prescribing HIV post-exposure prophylaxis for 28 days.

The need for post-exposure prophylaxis and reference and follow-up serum testing is determined by the victim’s immunity to HBV (vaccination status) and the (chance of) presence of HBV, HCV, or HIV in the source individual (e.g. known risk behaviour) [17]. When the source individual agrees to an (urgent) blood test, and the source tests negative, no serum needs to be taken from the employee, because no follow-up testing will be done. In case of an unknown, a positive or a source not willing to participate, reference and follow-up serology tests on the employee need to be performed by PHS. In case of a HIV positive or an unknown/unwilling source with possible HIV risk behaviour, the employee will be referred to the regional university hospital for risk assessment and prophylaxis.

### Analysis

A descriptive analysis was conducted to examine key variables related to OBEA, including workplaces, professions, age groups, types of injury, employees’ hepatitis B vaccination status, and post-exposure management including risk assessment.

#### Workplaces

OBEA cases were categorized per workplace (by most reported to least reported OBEA, with the two last groups being combined due to small numbers), including long-term care facilities, home care, mental health care, disability care, police departments, dental practices, ‘medical sterilization and other non-medical care (e.g. waste collection service), and ‘ambulance and other medical care’ (e.g. midwifery).

#### Professions

This study included the following professional categories: nurses, nurse assistants, nurses or nurse assistants (status unknown), medical doctors, police officers, mentors, nursing students, housekeeping staff, and medical assistants. Additionally, the category of ‘Others’ included employees working, for example, in dental practices or medical sterilization.

#### Age groups

Participants were grouped into three age categories: under 25 years, 25–50 years, and over 50 years.

#### Types of injury

Injuries were classified into the following categories: intramuscular needle, subcutaneous needle, lancets, venipuncture, instruments, biting, spitting/scratching, other, and unknown needle.

#### Employees hepatitis B vaccination

Employees were regarded as being fully vaccinated against hepatitis B if they had received at least three doses of the hepatitis B vaccine and had undergone an immune response assessment confirming vaccination efficacy. Individuals categorized as ‘not vaccinated’ included those who had received no vaccinations, were partially vaccinated, or were non-responders (HBV anti-bodies < 10IU/L).

#### Post-exposure management

Post-exposure management was based on the risk assessment (low-risk or high-risk OBEA) and categorized as no measures required, HBV immunization, source testing, collection of the employee’s reference serum, follow-up serum testing when needed, and administration of HIV post-exposure prophylaxis.

This approach allowed for a comprehensive analysis of OBEA cases across different professional groups and workplace settings, providing insights into risk profiles and management practices. To test for differences by profession and workplace on circumstances and post-exposure measures, we applied chi-square analyses and t-tests. Analyses were performed with SPSS 20.0 (IBM INC., Somers, NY, USA).

For readability, only in case of statistically significant results, P-values are mentioned in the text. A P-value <0.05 was considered statistically significant.

### Medical ethical statement

The Medical Ethics Committee of Maastricht University Medical Centre (MUMC+) exempted this study from official approval under prevailing laws in the Netherlands after official review of the study protocol as all data presented in this paper were retrospectively retrieved from regular infectious disease control activities and were deidentified, coded, and analysed anonymously (METC number: 2021-2901).

## Results

A total of 975 OBEA for non-hospital employees were included in the analyses (Table 1). The number of OBEA was consistent over time with about 115 incidents per year, with a decline within the last two years to approximately 90 OBEA per year.

**Table 1.** Sex and age distribution of occupational blood exposure accidents (OBEA) cases for different workplaces

Work setting		Long-term care facility	Home care	Mental health care	Disability care	Police departments	Dental practice	Medical sterilization and other non-medical care	Ambulance and other medical care
Total	N = 975	N = 491	N = 169	N = 74	N = 51	N = 50	N = 14	N = 68	N = 58
Men	166 (17%)	33 (7%)	12 (7%)	23 (31%)	7 (14%)	40 (80%)	1 (7%)	32 (47%)	18 (31%)
Women	809 (83%)	458 (93%)	157 (93%)	51 (69%)	44 (86%)	10 (20%)	13 (93%)	36 (53%)	40 (69%)
Age< 25	222 (23%)	128 (26%)	40 (24%)	15 (20%)	14 (27%)	2 (4%)	4 (29%)	12 (18%)	7 (12%)
25–50	572 (59%)	287 (59%)	101 (60%)	41 (55%)	27 (53%)	34 (68%)	8 (57%)	41 (60%)	33 (57%)
>50	128 (13%)	59 (12%)	26 (15%)	13 (18%)	7 (14%)	6 (12%)	0 (0%)	9 (13%)	8 (14%)
Age unknown	53 (5%)	17 (3%)	2 (1%)	5 (7%)	3 (6%)	8 (16%)	2 (14%)	2 (14%)	10 (17%)

### Workplaces

The majority (49%, N = 478) of OBEA were reported by long-term care facilities, followed by home care (17%, N = 166).

### Professions

Of all OBEA-reporting employees, 68% (N = 663) were working as nurses or nurse assistants.

Table 1 shows a further breakdown of the professions and workplaces. Overall, the vast majority of OBEA were reported by women (83% N = 809). In long-term care facilities 93% (N = 458) of the reports were done by female employees, while this was 20% (N = 10) at the police departments.

### Age groups

Of all employees, 25% were younger than 25 years old (n = 222), 61% were between 25 and 50 years old (n = 572), and 14% were older (>50 years old) (n = 128).

### Types of injury

**Figure 1.**
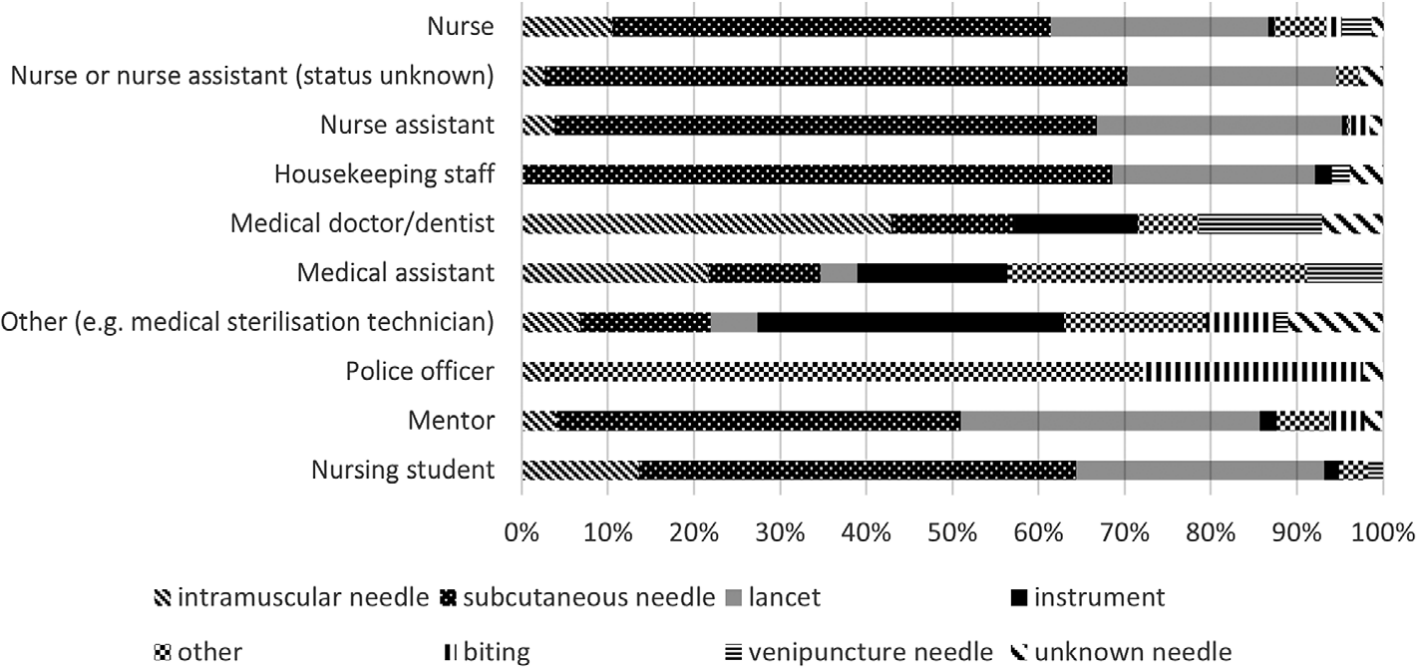
Percentage of types of exposure for the different professions.

The type of injury reported varied across professions as shown in Figure 1. Nurses, nurse assistants, nursing students, and housekeeping staff mainly reported subcutaneous needle (51–67%) and lancet (23–28%) injury; police officers reported bite incidents (26%) and spitting/scratching (70%); medical sterilization staff reported needle (11%) or instrument-related injuries (31%).

### Employees hepatitis B vaccination

The overall HBV vaccination coverage in OBEA reporting employees was 83% (n = 805) but varied between professions; it was 18% among housekeeping staff and up to 93% among medical doctors, police officers, nurses, and nurse assistants (Table 2).

**Table 2.** HBV vaccination coverage for the different professions and workplaces^a^

		Coverage and professions N = 975								
Profession	Nurses	Nurse assistants	Nurses or nurse assistants (status unknown)	Medical doctors	Police officers	Mentors	Nursing students	House-keeping staff	Medical assistants	Other^a^
Total OBEA (N)	285	341	37	14	43	49	59	51	23	73
Fully vaccinated (%)	92	89	89	93	91	76	86	18	87	51
Fully vaccinated (N)	263	303	33	13	39	37	51	9	20	37

		Coverage and workplaces N = 975								
	Long-term care	Home care	Police department	Disability care	Mental health care	Ambulance and other medical care	Dental practice	Medical sterilization and other non-medical care		
Total OBEA (N)	491	169	50	51	74	58	14	68		
Fully vaccinated (**%)**	86	83	90	78	84	91	93	40		
Fully vaccinated (N)	424	141	45	40	62	53	13	27		

a Other: employee working in dental practice or medical sterilization.

Coverage also varied between workplaces. Among ‘Ambulance and other medical care’ and the police departments, this was 90–93%, while within the disability care, home care, mental health care, and long-term care, 78–86% of affected individuals were fully vaccinated. Elderly workers (age >50 years) were less often vaccinated (76%) (P < 0.05).

Non-responders were observed in 1–3% of nurses, nurse assistants, and medical sterilization staff. Vaccination coverage increased over time, from 76% of all reporting employees being fully vaccinated and 20% unvaccinated in 2006 to 90% fully vaccinated and 9% unvaccinated in 2014. Vaccination coverage among nursing students has been consistently high with 100% since 2011.

### Post-exposure management

Of all reported OBEA, 419 (43%) were high-risk accidents. Within ‘Ambulance and other medical care’, ‘medical sterilization and other non-medical care’, police department and dental practice of all OBEA, more than 50% was assessed as high risk. Low-risk OBEAs were mostly seen among housekeeping staff (73%, N = 37) and nurse assistants (67% of all reporting nurse assistants, N = 228).

For more than half (52%, N = 503) of the reported OBEA, post-exposure measures were taken, comprising 392 high-risk and 111 low-risk accidents (78% and 22%, respectively).

The majority of post-exposure measures were carried out among medical assistants, while the fewest were conducted among nurses and nurse assistants (Table 3).

**Table 3.** Post-exposure measures for the different professions

Profession	Nurses	Nurse assistants	Nurses or nurse assistants (status unknown)	Medical doctors	Police officers	Mentors	Nursing students	House-keeping staff	Medical assistants	Other^a^
Post-exposure measures taken N (%)	137 (48)	13 (35)	141 (41)	11 (79)	26 (60)	30 (61)	30 (51)	40 (78)	19 (83)	56 (77)
Total OBEA (N = 975)	285	37	341	14	43	49	59	51	23	73

a Other: employee working in dental practice or medical sterilization.

An overview of risk assessment and post-exposure measures is depicted in Table 4. This table shows the total number of low- and high-risk OBEA reported, during the time period 2006–2014, by workplace and profession.

**Table 4.** Total number of low and high risk OBEA reported, 2006–2014, by workplace and profession (N = 975)

		Low risk OBEA N = 556 (57%)					High risk OBEA N = 419 (43%)				
Workplace Total OBEAs	Profession	OBEA	Fully HBV- vaccinated^a^	Source known	Source testing	Post-exposure measures	OBEA	Fully HBV-vaccinated^a^	Source known	Source testing	Post-exposure measures
		N(%)	N(%)	N(%)	N(%)	N(%)^a^	N(%)	N(%)	N(%)	N(%)	N(%)^a^
Long-term care facility (N = 491)	Nurses and nurse assistants	254 (16 .3)	232 (91.3)	166 (65.4)	21 (8.3)	11 4.3)	146 (36.5)	127 (87.0)	130 (89)	120 (82.2)	27 (18.5)
	Nursing students	22 (62.9)	19 (86.4)	17 (77.3)	3 (13.6)	2 (9.1)	13 (37.1)	13 (100)	10 (76.9)	10 (76.9)	2 (15.4)
	Housekeeping staff	12 (66.7)	4 (33.3)	10 (83.3)	8 (66.7)	1 (8.3)	6 (33.3)	1 (16.7)	5 (83.3)	5 (83.3)	1 (16.7)
	Other	22 (57.9)	18 (81.8)	14 (63.6)	2 (9.1)	3 (13.6)	16 (42.1)	10 (62.5)	12 (75)	12 (75)	4 (25)
Home care (N = 169)	Nurses and nurse assistants	80 (64.5)	74 (92.5)	49 (61.3)	6 (7.5)	5 (6.3)	44 (35.5)	42 (95.5)	44 (100)	36 (81.8)	2 (4.5)
	Nursing students	6 (60)	6 (100)	2 (33.3)	0 (0)	0 (0)	4 (40)	4 (100)	4 (100)	4 (100)	0 (0)
	Housekeeping staff	15 (71.4)	2 (13.3)	14 (93.3)	11 (73.3)	1 (6.7)	6 (28.6)	0 (0)	6 (100)	5 (83.7)	0 (0)
	Other	9 (64.3)	8 (88.9)	4 (44.4)	0 (0)	0 (0)	5 (35.7)	5 (100)	5 (100)	3 (60)	0 (0)
Mental health care (N = 74)	Nurses and nurse assistants	26 (48.1)	25 (96.2)	14 (53.8)	2 (7.7)	1 (3.8)	28 (51.9)	22 (78.6)	25 (89.3)	24 (85.7)	8 (28.6)
	Housekeeping staff	2 (66.7)	1 (50)	1 (50)	0 (0)	0 (0)	1 (33.3)	0 (0)	0 (0)	0 (0)	1 (100)
	Mentors	6 (60)	3 (50)	3 (50)	2 (33.3)	3 (50)	4 (40)	4 (100)	3 (75)	2 (50)	2 (50)
	Other	4 (57.1)	4 (100)	3 (75)	0 (0)	1 (25)	3 (42.9)	3 (100)	3 (100)	2 (66.7)	1 (33.3)
Disability care (N = 51)	Nurses and nurse assistants	9 (75)	7 (77.8)	6 (66.7)	2 (22.2)	0 (0)	3 (25)	3 (100)	3 (100)	3 (100)	0 (0)
	Mentors	21 (58.3)	15 (71.4)	13 (61.9)	5 (23.8)	2 (9.5)	15 (41.7)	13 (86.7)	14 (93.3)	11 (73.3)	4 (26.7)
	Other	3 (100)	2 (66.7)	3 (100)	0 (0)	1 (33.3)	0 (0)	–	–	–	–
Ambulance and other medical care (N = 58)	Nurses	8 (27.6)	7 (87.5)	5 (62.5)	0 (0)	1 (12.5)	21 (72.4)	21 (100)	15 (71.4)	8(38.1)	14 (66.7)
	Medical doctors	1 (10)	1 (100)	0 (0)	0 (0)	0(0)	9 (90)	8 (88.9)	6 (66.7)	6 (66.7)	3 (33.3)
	Other	4 (21.1)	4 (100)	3 (75)	1 (25)	0 (0)	15 (78.9)	12 (80)	11 (73.3)	8 (53.3)	8 (53.3)
Police department (N = 50)	Police officers	20 (48.8)	18 (90)	15 (75)	1 (5)	5 (25)	21 (51.2)	20 (95.2)	18 (85.7)	15 (71.4)	5 (23.8)
	Other	4 (44.4)	4 (100)	3 (75)	0(0)	0 (0)	5 (55.6)	3 (60)	5 (100)	4 (80)	2 (40)
Medical sterilization and other non-medical care (N = 68)	Nurses	1 (16.7)	1 (100)	0 (0)	0 (0)	0 (0)	5 (83.3)	4 (80)	3 (60)	2 (40)	3 (6)
	Housekeeping staff	7 (87.5)	1 (14.3)	2 (28.6)	0 (0)	6 (85.7)	1 (12.5)	0 (0)	0 (0)	0 (0)	1 (100)
	Technicians/ other	19 (35.2)	4 (21.1)	2 (10.5)	2 (10.5)	11 (57.9)	35 (64.8)	17 (48.6)	5 (14.3)	3 (8.6)	30 (85.7)
Dental practice (N = 14)	Medical Assistants	1 (8.3)	1 (100)	0 (0)	0 (0)	1 (100)	11 (91.7)	10 (90.9)	7 (63.6)	5 (45.5)	6 (54.5)
	Other	0 (0)	–	–	–	–	2 (100)	2 (100)	2 (100)	2 (100)	0(0)
	Total (N)	555	461	349	66	55	416	344	336	290	124

a Employee was fully HBV vaccinated before reporting OBEA.

Regarding HBV immunization/post-exposure prophylaxis, hepatitis B immunoglobulins (total N = 37) were mainly administered in OBEA in housekeeping and medical sterilization staff, involving 16% of all housekeeping staff (N = 8) and 21% of all medical sterilization staff (N = 14). This is, respectively, 22% and 38% of total amount of immunoglobulins given.

HBV vaccination (total N = 62) was initiated for 33% of all medical sterilization staff (N = 22), 24% of housekeeping staff (N = 12), and 23% of mentors (N = 11).

Of all employees whose serum was taken (N = 137), post-exposure follow-up serum testing was conducted for 122 employees, more frequently among household staff (18% of all staff, N = 9), police officers (21% of all officers, N = 9), and medical sterilization staff/other non-medical care (49% of all staff, N = 36). Within 6 months, none of the employees seroconverted.

For 685 reported OBEA (70%), the source was identified, being 349 low-risk (63% of all low-risk accidents) and 336 high-risk accidents (80%). Source identification was notably high among police officers and nurse assistants (both 81%).

Source testing was conducted in 356 OBEA (37% of all reported accidents), with higher testing rates among police officers, medical assistants, and housekeeping staff (42–58% of reporting profession) and lower rates among medical sterilization staff (15% of all medical sterilization staff).

Overall, five sources tested positive for HBsAg (prevalence 1.4%) and seven for anti-HCV (2.0%). A positive HIV source was identified in 10 OBEA notifications (2.8%). However, seven simultaneous low-risk OBEA (among police officers) were linked to the same positive HIV source. In total, four unique sources tested HIV positive (prevalence 1.1%). Regarding one OBEA notification, the source tested positive for both HBV and HCV, while another one tested positive for both HBV and HIV.

HIV post-exposure prophylaxis (PEP) was provided to three employees working in police department, ambulance transport, and mental health care. See Table 5.

**Table 5.** Post-exposure measurements taken in low- and high-risk OBEA

Measures (N)	Low-risk OBEA (N = 556)				High-risk OBEA (N = 419)			
	HBV vaccination status employee				HBV vaccination status employee			
	Fully vaccinated (461/83%)	Not fully vaccinated (93/17%)	Not responding (2/<1%)	Total	Fully vaccinated (344/82%)	Not fully vaccinated (69/17%)	Not responding (6/1%)	Total
Source tested	11	53	2	66	250	36	4	290
Source HIV positive (N)	7	0	0	7	4^a^	1	0	5^a^
Source HCV positive (N)	1	0	0	1	6^a^	1	0	7^a^
Source HBV positive (N)	0	0	0	0	4^a^	0	0	4^a^
Employees serum token (N)	6	15	0	21	83	30	3	116
Offered HBV vaccination (N)	0	33	0	33	0	29	0	29
HBIg administration (N)	0	12	0	12	0	22	3	25
HIV-PEP (N)	0	0	0	0	2	1	0	3

a Of all tested persons, we found two double infections among the sources (1 HBV/HCV pos and 1 HBV/HIV pos).

Notably, a relatively high percentage of sources were tested among housekeeping staff despite their involvement in low-risk OBEA.

## Discussion

Our findings provide a comprehensive overview of the frequency, circumstances, and risk factors associated with occupational blood exposure accidents (OBEA) in non-hospital settings, highlighting critical gaps in preventive measures and post-exposure management across various professional groups. With 975 OBEA cases reported over 9 years, the data from this large study reveal significant variation in HBV vaccination coverage, types of injury, and post-exposure management across different professions and workplaces. Remarkable findings included the overall low hepatitis B vaccination coverage, the relatively low vaccination rate among older employees, and the many accidents reported by nurses and nurse assistants caused by insulin needles, insulin pens, and glucose meter needles and lancets. The results of our study emphasize the persistent vulnerability of non-hospital professionals. Outcomes also underscore the need for targeted strategies to improve preventative measures like vaccination and mitigate OBEA risks.

A striking finding was the substantial variation in hepatitis B vaccination coverage, which was generally low. This led to an increased need for post-exposure measures in low-risk OBEA, which would not be necessary with a high vaccination rate. The average vaccination rate for all employees included in this study was 80%, ranging from 18% to over 90%, despite the rollout of national vaccination guidelines and occupational immunization programmes. Vaccination rates were the lowest in housekeeping staff and highest among nurses, nurse assistants, and police officers, but among them still, nearly 10% were not fully vaccinated. Only among nursing students an increase up to 100% in vaccination coverage was observed, showing the positive results of offering vaccination during education. In contrast, older employees (>50years) were generally more often not vaccinated.

Most countries have incorporated HBV vaccination in their national immunization programmes for all children. In the Netherlands, universal hepatitis B vaccination was introduced in 2011 for children born on or after August of that year. For a longer period, occupational HBV vaccination strategies have been in place for all health care workers who may have direct contact with patients’ blood, blood-stained body fluids, or tissues [27].

Based on our study results, all employees at workplaces with a reasonable risk of exposure to blood should be offered hepatitis B vaccination (e.g. housekeeping staff).

However, employee mobility, particularly in home care settings, remain a challenge for achieving full HBV-B coverage. Workplaces should therefore be encouraged to develop an action plan for all workers, with particular attention to those above 50 years old, to support successful vaccination programme implementation.

Another noteworthy finding was the difference in occurrence and characteristics of OBEA among workplaces and professions. Housekeeping staff (in a healthcare setting) appeared to be at risk for OBEA, primarily experiencing low-risk accidents, relatively often with unknown needles. At the same time, they had the lowest HBV vaccination coverage among all groups (18%). As a result, they often required post-exposure measures that would have been unnecessary if they were properly immunized. It is essential to take actions to improve their HBV vaccination status. Additionally, housekeeping staff within healthcare should receive education and training on OBEA awareness and injury prevention [28].

Based on our study findings, mentors working in disabled care centres are also advised to increase their HBV vaccination coverage, which stood at 76%.

For employees working at medical sterilization, the observed low HBV vaccination rate (51%) requires attention as well. Mainly high-risk accidents were reported, often with an unknown source since medical instruments mostly cannot be traced back, which meant that post-exposure reference and follow-up serum testing was needed regularly.

Serum testing was more often needed for police officers because the source did not always agree (right away) to undergo serology testing. Prevalence of HBV, HCV, and HIV in detainees is higher than in the general population due to risk behaviour such as injecting drug use and unsafe sex, coupled with pre-detention social vulnerability [29, 30]. Next to HBV vaccination, training police officers to avoid exposures remains the most critical measure for reducing transmission risks [22].

Notable was the finding that a high proportion of nurses and nurse assistants reported accidents, some categorized as high-risk, specifically caused by insulin needles, insulin pens, and glucose meter needles and lancets. A possible explanation might be that nurses and nurse assistants more often perform routine procedures using medical devices linked to OBEA-risk, for example, giving an insulin injection. Kiss et al. found insulin pens indeed being the most frequent cause of NSI among nursing personnel and often being connected with unsafe needle-handling practices [14]. Therefore, preventive actions for nurses and nurse assistants should focus on strategies specifically avoiding these types of injuries.

In our study, the chance that a source tested positive proved to be small. Only a few source individuals tested positive for HBV, HCV, or HIV. This fits the expectation, since, in the Netherlands, the seroprevalence of HBV, HCV, and HIV is generally low. The estimated Dutch HBV prevalence is 0.2%, for HCV this is 0.1–0.4% [23, 24]. The prevalence of HIV infection in the general population is also low (0.2%) but higher in specific sub-populations [25]. Only a few infections are related to blood exposure accidents in the Netherlands [11]. This was confirmed by our study where none of the tested employees seroconverted. It appears reasonable to consider in countries with low HBV prevalence, unnecessary source testing can be minimized by omitting tests for low-risk exposure incidents among fully vaccinated, healthy healthcare workers, while reserving testing for high-risk exposures on a case-by-case basis.

Over the last decade, the international migration of health workers has increased [31] from countries in Eastern Europe, Asia, and Africa, where the prevalence of chronic HBV, as well as HCV and HIV, is considerably higher than in western/high-income countries. Globally among health care workers, the prevalence of acute hepatitis B infection is 5.3%, being more prevalent in health care workers in low-income countries, particularly in Africa, whereas the highest immunization rates against HBV in health care workers were recorded in high-income countries. [32]. It is suggested that policies for screening and vaccinating health care workers be reviewed and, where appropriate, updated in line with the existing HBV vaccination guidelines in the Netherlands.

Another point of attention is the increasing number of immunocompromised workers taking **immunosuppressive drugs** for the management of autoimmune inflammatory conditions, who were not addressed in this study [33]. This enables them to participate better in daily activities including work but might raise concerns regarding the immune response to HBV vaccination (non-responding) and risk for infectious diseases at the workplace. It is important that employers are aware of possibly having more non-responders at their workplace. Non-responders should not delay notifying an OBEA, since for them immediate action might be required including administering hepatitis B immunoglobulins.

One limitation of our study was the difficulty in addressing the prevalence of OBEA outside hospital settings due to insufficient data on the entire population of employees served (denominator) from which our sample of accident notifications originated. A large part of this population consisted of around 11,500 employees from long-term care facilities, home care, and police who were vaccinated by PHS South-Limburg over a period of 13 years including our study time frame [26]. This number would yield a crude OBEA prevalence estimate of 0.5%. The PHS did not exclusively receive accident notifications from the institutions mentioned above, so most likely, the total extent of its blood-borne accident care is probably larger.

Another limitation was the lack of consistent data on the specific work procedures during which the accidents occurred in some cases. However, on the accident registration forms, activities concerning the needle waste container, recapping of needles, police arrests, unexperienced nursing students performing various medical procedures, and needles left in inappropriate places such as waste or bed were regularly noted. These are valuable observations when developing customized prevention guidance.

In addition, although the data set is from some years ago, we expect the situation has not much changed. Given the richness of the dataset and the inclusion of various non-hospital workplaces, this study provides valuable outcomes. Although the data were collected from a single region, it likely reflects broader patterns across the Netherlands.

Generally, OBEA outside hospital settings are known to be largely under-reported and, thus, causing a bias in outcomes [12, 13]. Unlike in hospitals, monitoring systems to record accidents are often not in place [15]. Even when reported internally at work, accidents might not be notified to a central office. High-risk OBEA needing post-exposure measures would rather be notified than low-risk accidents, leading to an over-representation of high-risk OBEA. The slight decrease in the number of accident notifications received in 2013 and 2014 compared with previous years could be related to the EU directive that came into force by the end of 2013 and required the use of safety needles [13, 34]. Raised awareness and knowledge among employees could also play a role. However, it is still important not to underestimate the seriousness of NSI [35]. Despite the implementation of EU regulations, O’Sullivan reported in 2020 no reduction in NSI but a lower incidence of NSI from ‘disposable needles with syringes’ after the implementation of the EU regulations [4].

In contrast to the current generic OBEA protocols, the high diversity in OBEA as shown by this study emphasizes the necessity of turning to profession- and workplace-specific preventive actions [36]. This will enhance prevention and will lead to adequate post-exposure measures. While previous studies by Chen and Schneeberger suggested this as well, implementation of such targeted prevention remains a challenge [36, 37].

## Conclusion

Employees outside the hospital are at risk of NSI and other OBEA. The – albeit increasing – insufficient vaccination rate and the frequent occurrence of OBEA highlight the need for sustained attention from both employees and employers, with a focus on addressing the specific issues within each occupational group, for both health care workers and non-health care workers.

Our study findings underscore the importance of offering more tailored recommendations for the prevention and management of OBEA, considering their unique circumstances within different professions and workplaces. Occupational health services must continue to play a vital role in optimizing the provision of occupational hepatitis B vaccinations. Vaccinating nursing students during their education has proven to be an effective strategy, resulting in high levels of protection within this group.

This study also raises an important consideration regarding the management of low-risk accidents. Given the extremely low number of positive sources, it may be worth revisiting the necessity of initiating follow-up procedures for certain low-risk accidents after reporting an OBEA in countries with low HBV prevalence. For fully vaccinated employees involved in low-risk incidents, it may be reasonable to forgo post-exposure measures, given the overall low risk of OBEA in settings like the Netherlands.

It remains important to notify and register OBEA in a standardized way in order to keep track of occurrence, risk groups, and circumstances of these accidents. In this way, OBEA prevention and post-exposure measures can be evaluated and OBEA protocols can be adapted accordingly.

## Supporting information

10.1017/S0950268826101319.sm001 Brouwers et al. supplementary material Brouwers et al. supplementary material

## Data Availability

The data sets used and analysed during the current study are available from the corresponding author on reasonable request.

## Abbreviations

BBV: blood-borne virus
EU: European Union
HBV: hepatitis B virus
HCV: hepatitis C virus
HCW: health care workers
HIV: human immunodeficiency virus
NSI: needle stick injuries
OBEA: occupational blood exposure accidents
PHS: Public Health Service
WHO: World Health Organization

## Supplementary material

The supplementary material for this article can be found at http://doi.org/10.1017/S0950268826101319.

## References

[1] Auta A, et al. (2018) Global prevalence of percutaneous injuries among healthcare workers: A systematic review and meta-analysis. International Journal of Epidemiology 47, 1972–1980. 30272173 10.1093/ije/dyy208

[2] De Perio M, Victory K and Groenewold M (2018) Needlestick injuries and other potential exposures to bloodborne pathogens among police officers in a city police department, 2011–2016. Open Forum Infectious Diseases 26 (Suppl 1), S348.

[3] Brouillette NM, Quinn MM and Kriebel D (2017) Risk of sharps injuries to home care nurses and aides: A systematic review and meta-analysis. Journal of Occupational and Environmental Medicine 59, 1072 –1077. 28930800 10.1097/JOM.0000000000001160 PMC5671783

[4] O’Sullivan G and Gallagher J (2020) Have legislative interventions impacted the incidence of needlestick injuries? Irish Medical Journal 112, 1023. 32311253

[5] Reddy VK, et al. (2017) Devices for preventing percutaneous exposure injuries caused by needles in healthcare personnel. Cochrane Database of Systematic Reviews 11, Cd009740. 29190036 10.1002/14651858.CD009740.pub3 PMC6491125

[6] Kofman AD, et al. (2025) US Public Health Service guidelines for the management of occupational exposures to human immunodeficiency virus and recommendations for post-exposure prophylaxis in healthcare settings. Infection Control and Hospital Epidemiology 46(9), 863–873. 10.1017/ice.2025.10254. 41569270 PMC12616222

[7] Pintilie H, Brook G. (2018) Commentary: A review of risk of hepatitis B and C transmission through biting or spitting. Journal of Viral Hepatitis 25, 1423–1428. 30047616 10.1111/jvh.12976

[8] Cresswell FV, et al. (2018) A systematic review of risk of HIV transmission through biting or spitting: Implications for policy. HIV Medicine 19, 532–540. 29687590 10.1111/hiv.12625 PMC6120498

[9] Public Health England (2019) Guidance on Management of Potential Exposure to Blood-Borne Viruses in Emergency Workers, O’Moore É, et al. (eds.). London: Department of Health and Social Care.

[10] Vermeiren AP, Hoebe CJ, Dukers-Muijrers NH. (2013) High non-responsiveness of males and the elderly to standard hepatitis B vaccination among a large cohort of healthy employees. Journal of Clinical Virology 58, 262–264. 23895931 10.1016/j.jcv.2013.07.003

[11] Prüss-Ustün A, Rapiti E, Hutin Y. (2005) Estimation of the global burden of disease attributable to contaminated sharps injuries among health-care workers. American Journal of Industrial Medicine 48, 482–490. 16299710 10.1002/ajim.20230

[12] WHO (World Health Organization) (2024) Occupational infections: The most common occupational infections of concern in the health sector are tuberculosis, hepatitis B and C, HIV/AIDS and respiratory infections (coronaviruses, influenza). Available at https://www.who.int/publications/i/item/9789240052154 (accessed 8 December 2025).

[13] HOSPEEM, EPSU (European Federation of Public Services Unions). (2009) Framework agreement on prevention from sharp injuries in the hospital and healthcare. Available at https://www.epsu.org/article/framework-agreement-prevention-sharp-injuries-hospital-and-health-care-sector (accessed 8 December 2025).

[14] Mengistu DA, Tolera ST, Demmu YM. (2021) Worldwide prevalence of occupational exposure to needle stick injury among healthcare workers: A systematic review and meta-analysis. Canadian Journal of Infectious Diseases and Medical Microbiology 2021, 1–10. 10.1155/2021/9019534 PMC7864758 33564345

[15] Bouya S, et al. (2020) Global prevalence and device related causes of needle stick injuries among health care workers: A systematic review and meta-analysis. Annals of Global Health 86, 35. 32346521 10.5334/aogh.2698 PMC7181946

[16] Ministry of Social Affairs and Employment. (1998) Working Conditions Act 1998 (Arbeidsomstandighedenwet 1998). The Netherlands/ The Hague.

[17] van Wijk PT, et al. (2010) Occupational blood exposure accidents in the Netherlands. European Journal of Public Health 20, 281–287. 19864365 10.1093/eurpub/ckp163

[18] Vos D, Götz HM, Richardus JH. (2006) Needlestick injury and accidental exposure to blood: The need for improving the hepatitis B vaccination grade among health care workers outside the hospital. American Journal of Infection Control. 34, 610–612. 17097460 10.1016/j.ajic.2006.02.004

[19] Gershon RR, et al. (2007) Non-hospital based registered nurses and the risk of bloodborne pathogen exposure. Industrial Health 45, 695–704. 18057813 10.2486/indhealth.45.695

[20] Hatzakis A, et al. (2020) Securing sustainable funding for viral hepatitis elimination plans. Liver International. 40, 260–270. 31808281 10.1111/liv.14282

[21] Eaton J, et al. (2023) The negative impact of global health worker migration, and how it can be addressed. Public Health 225, 254–257. 37949017 10.1016/j.puhe.2023.09.014

[22] Kremer M., et al. (2022) A Carefully Considered Labour Migration Policy. How Long-Term Care can Benefit from Skilled Migrants. The Hague: The Dutch Advisory Council on Migration (Adviesraad Migratie).

[23] Kiss P, de Meester M, Braeckman L. (2008) Needlestick injuries in nursing homes: The prominent role of insulin pens. Infection Control and Hospital Epidemiology. 29, 1192–1194. 18950277 10.1086/592407

[24] van Wijk PT, et al. (2006) Differences between hospital- and community-acquired blood exposure incidents revealed by a regional expert counseling center. Infection 34, 17–21. 16501897 10.1007/s15010-006-4125-9

[25] Schneeberger PM, et al. (2011) Risk-Estimation and Handling of Occupational Blood Exposure Accidents by Nationally Operating Telephone Service in the Netherlands. BMC Proceedings. 5 (Suppl. 6): P216.

[26] LCI (National Coordination Centre for Infectious Disease Control). (2019) National Dutch guideline on needlestick incidents. Available at https://lci.rivm.nl/richtlijnen/prikaccidenten (accessed 8 December 2025).

[27] WHO (World Health Organization). (2022) Implementation guide for vaccination of health workers.

[28] Cheetham S, et al. (2021) Education and training for preventing sharps injuries and splash exposures in healthcare workers. Cochrane Database of Systematic Reviews 4, Cd012060. 33871067 10.1002/14651858.CD012060.pub2 PMC8094230

[29] Barreira-Díaz A, Buti M. (2021) Prisons: An important link in the elimination of hepatitis B. *Revista Española de Sanidad Penitenciari*a 23, 88–90. 35411917 10.18176/resp.00036 PMC8802823

[30] Falla AM, et al. (2018) Hepatitis B/C in the countries of the EU/EEA: A systematic review of the prevalence among at-risk groups. BMC Infectious Diseases 18, 79. 29433454 10.1186/s12879-018-2988-x PMC5809955

[31] Drennan VM, Ross F. (2019) Global nurse shortages-the facts, the impact and action for change. British Medical Bulletin 130, 25–37. 31086957 10.1093/bmb/ldz014

[32] Mahamat G, et al. (2021) Global prevalence of hepatitis B virus serological markers among healthcare workers: A systematic review and meta-analysis. World Journal of Hepatology 13, 1190–1202. 34630885 10.4254/wjh.v13.i9.1190 PMC8473496

[33] Azoulay E, et al. (2020) Diagnosis of severe respiratory infections in immunocompromised patients. Intensive Care Medicine 46, 298–314. 32034433 10.1007/s00134-019-05906-5 PMC7080052

[34] Lu Y, et al. (2015) Effectiveness of safety-engineered devices in reducing sharp object injuries. Occupational Medicine 65, 39–44. 25344960 10.1093/occmed/kqu152

[35] Brusini A. (2022) Needle stick injuries among nurses in Italy: A review. Giornale Italiano Medicina del Lavoro ed Ergonomia 44, 391–396. 36622828

[36] Schneeberger PM, et al. (2012) Registration of blood exposure accidents in the Netherlands by a nationally operating call center. Infection Control & Hospital Epidemiology 33, 1017–1023. 22961021 10.1086/667728

[37] Chen GX, Jenkins EL. (2007) Potential work-related exposures to bloodborne pathogens by industry and occupation in the United States part II: A telephone interview study. American Journal of Industrial Medicine 50, 285–292. 17340611 10.1002/ajim.20441

