# Supplementary material for: Non-hospital occupational blood exposure accidents: A nine-year retrospective analysis of post-exposure HIV, hepatitis B and C risk management in the Netherlands
Source: Epidemiol Infect. 2026 Mar 27;154:e58. doi: 10.1017/S0950268826101319 (PMC13161801; doi:10.1017/S0950268826101319)

Appendix 1. Summary of the (Dutch) National Guideline for Needle Stick Accidents


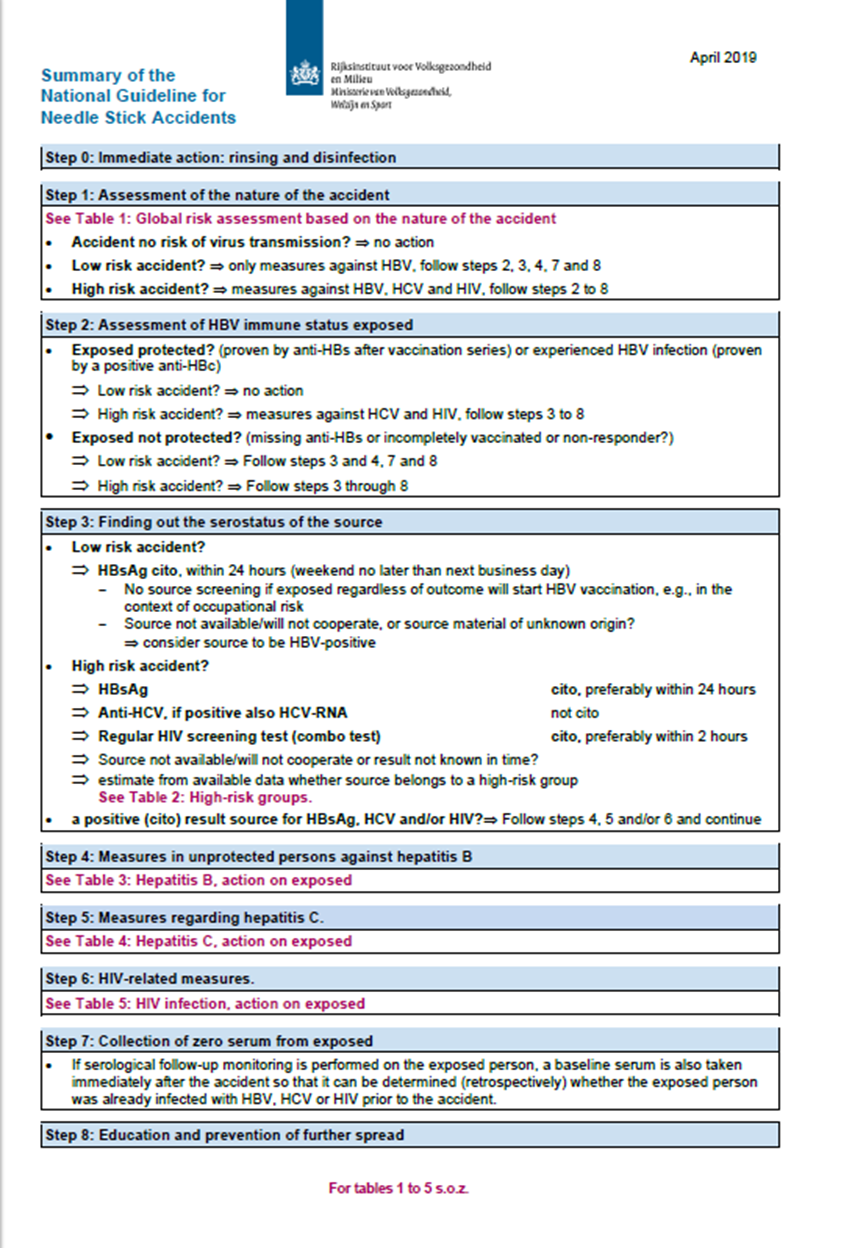


Table 1: Global risk assessment based on the nature of the accident
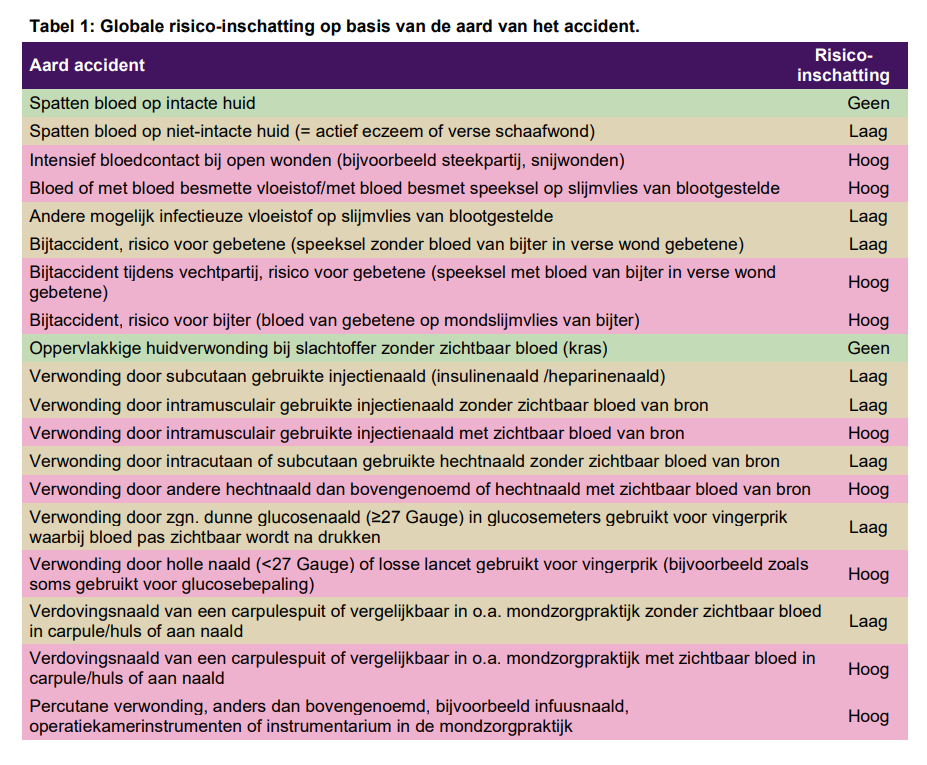


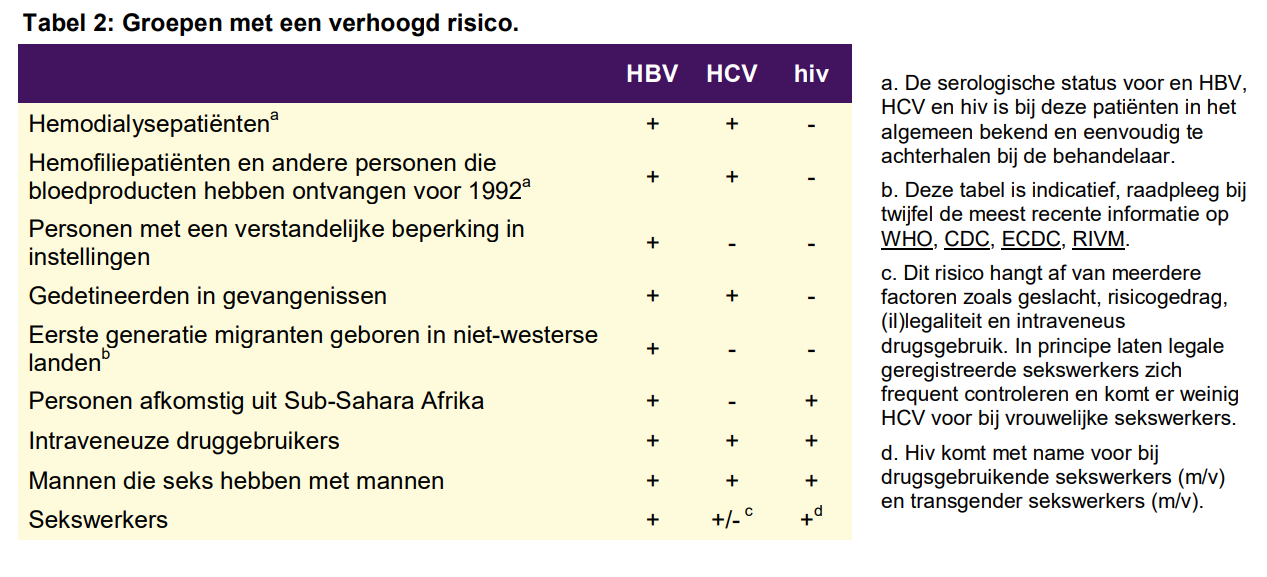
Table 2: Groups with an increased risk.


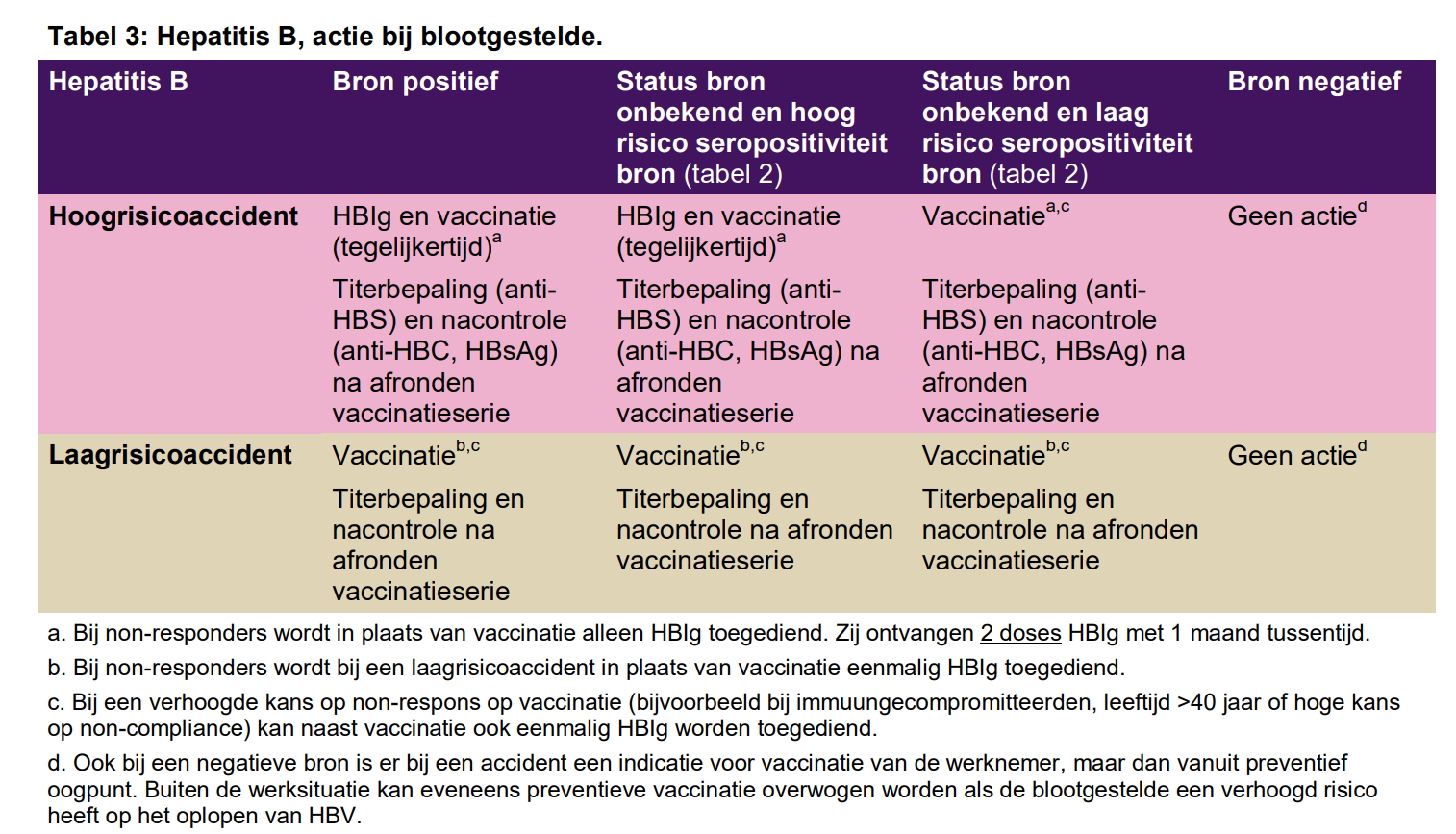
Table 3: Hepatitis B, actions for the exposed person


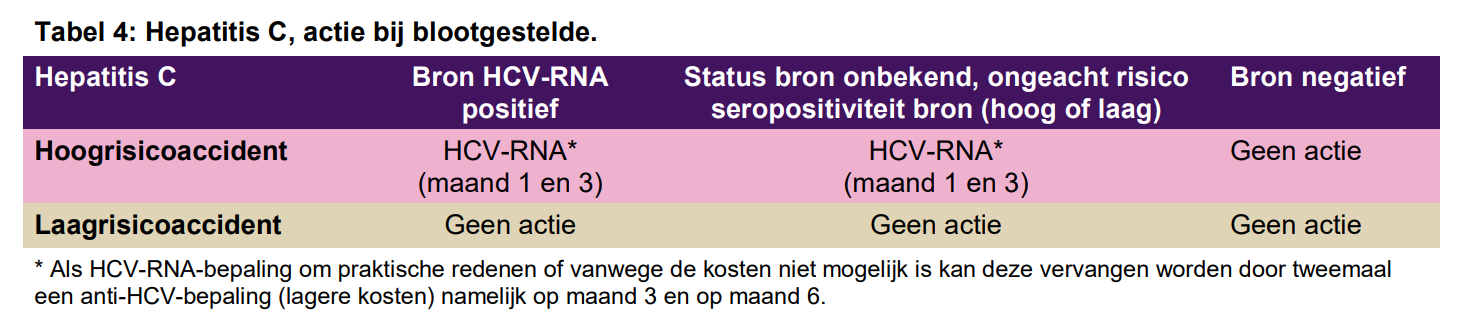
Table 4: Hepatitis C, actions for the exposed person

Table 5: HIV infection, actions for the exposed person.


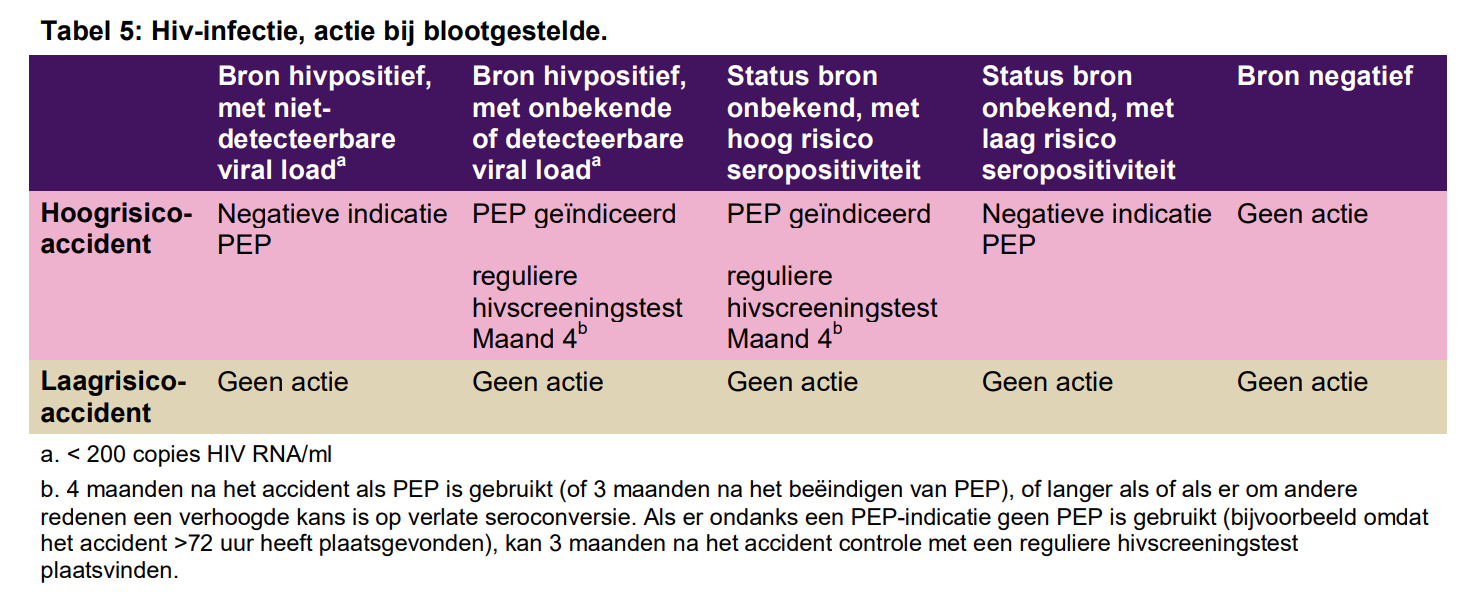

Supplement: Brouwers et al. supplementary material [file S0950268826101319sup001.docx]
